# Investigating the influence of elevated temperature on nutritional and yield characteristics of mung bean (*Vigna radiata* L.) genotypes during seed filling in a controlled environment

**DOI:** 10.3389/fpls.2023.1233954

**Published:** 2023-09-21

**Authors:** Manu Priya, Anjali Bhardwaj, Uday Chand Jha, Bindumadhava HanumanthaRao, P. V. Vara Prasad, Kamal Dev Sharma, Kadambot H.M. Siddique, Harsh Nayyar

**Affiliations:** ^1^ Department of Botany, Panjab University, Chandigarh, India; ^2^ ICAR-Indian Institute of Pulses Research, Kanpur, India; ^3^ Department of Agronomy and Sustainable Intensification Innovation Lab, Kansas State University, Manhattan, KS, United States; ^4^ Dr. Marri Channa Reddy Foundation, Hyderabad, Telangana, India; ^5^ Department of Agricultural Biotechnology, Chaudhary Sarwan Kumar (CSK) Himachal Pradesh Agricultural University, Palampur, India; ^6^ The UWA Institute of Agriculture, The University of Western Australia, Perth, WA, Australia

**Keywords:** heat stress, legumes, pulses, grains, seed quality, proteins, yield

## Abstract

Rising temperatures impact different developmental stages of summer crops like mung bean, particularly during the crucial seed-filling stage. This study focused on two mung bean genotypes, categorized as heat-tolerant [HT] or heat-sensitive [HS]. These genotypes were grown in pots in an outdoor natural environment (average day/night temperature 36°C/24.3°C) until the onset of podding (40 days after sowing) and subsequently relocated to controlled-environment walk-in growth chambers for exposure to heat stress (42°C/30°C) or control conditions (35°C/25°C) until maturity. For all measured attributes, heat stress had a more pronounced effect on the HS genotype than on the HT genotype. Heat-stressed plants exhibited severe leaf damage, including membrane damage, reduced chlorophyll content, diminished chlorophyll fluorescence, and decreased leaf water content. Heat stress impeded the seed-filling rate and duration, decreasing starch, protein, fat, and mineral contents, with a notable decline in storage proteins. Heat stress disrupted the activities of several seed enzymes, inhibiting starch and sucrose accumulation and consequently decreasing individual seed weights and seed weight plant^−1^. This study revealed that heat stress during seed filling severely impaired mung bean seed yield and nutritional quality due to its impact on various stress-related traits in leaves and enzyme activities in seeds. Moreover, this research identified potential mechanisms related to heat tolerance in genotypes with contrasting heat sensitivity.

## Introduction

The gradual increase in global average global temperatures, especially in tropical and subtropical regions, poses significant challenges to warm-season food crops (Jha et al., 2014), such as mung beans, compromising their yield and nutritional security (Bhardwaj et al., 2023). Crops thrive within specific temperature ranges (minimum and maximum), with those exceeding the maximum considered heat stress (Wahid et al., 2007; Hasanuzzaman et al., 2013; Kumari et al., 2021). Heat stress triggers various morphological, anatomical, physiological, biochemical, and molecular changes in leaves, flowers, and seeds, thereby curtailing the overall growth and yield (Bita and Gerats, 2013). Manifestations include leaf and stem scorching, leaf abscission, senescence of leaves, flowers, and fruiting structures, and reduced growth of leaves, shoots, roots, and seeds (Vollenweider and Günthardt-Goerg, 2005; Chaudhary et al., 2022b).

Heat stress profoundly affects the reproductive stage of plants, leading to flower and fruiting structure abortion, as observed in cereals (Niu et al., 2021) and legumes (Liu et al., 2019; Basu et al., 2022). Seed filling plays a crucial role in determining the seed weight and yield (Devasirvatham et al., 2012; Jiang et al., 2015). This process involves the transportation of precursor molecules and minerals from leaves to seeds to synthesize storage constituents, such as carbohydrates, fats, and proteins (Barnabás et al., 2008; Awasthi et al., 2014). Abiotic stresses, such as heat, negatively impact leaf function, flowering, seed development, and seed composition in legumes (Sarkar et al., 2021), causing significant yield losses (Kumar et al., 2021).

Mung bean (*Vigna radiata* (L.) R. Wilczek) is the second most cultivated leguminous crop after chickpea and is grown on over six million hectares globally. India contributes approximately 54% of the global mung bean production (HanumanthaRao et al., 2016; Nalajala et al., 2023). Mung bean seeds have high nutritional value, containing 22%–27% protein; essential vitamins such as B5, B6, thiamine, and niacin; minerals such as magnesium and iron; and dietary fiber. Mung bean seeds also contain beneficial compounds, such as alkaloids, coumarins, phytosterins, polyphenols, oligosaccharides, and antioxidants, which support physiological metabolism in animals and humans (Tang et al., 2014). Mung beans have low input requirements, are adaptable, and are short-duration grain legumes (65–90 days) (HanumanthaRao et al., 2016).

Mung beans are primarily grown in tropical areas characterized by dry or semiarid conditions, usually during fall and summer. Their ideal temperature range is 27°C–30°C (Pannu and Singh, 1993). However, as a warm-season crop, mung beans are susceptible to extreme heat (Sharma et al., 2016), impairing vegetative and reproductive growth (HanumanthaRao et al., 2016).

Although the adverse impact of heat stress on overall plant growth and yield is well established (Sharma et al., 2016), little is known about its influence on pod formation, seed filling, seed size, and seed quality in mung bean. Therefore, this study aimed to determine the detrimental effects of heat stress during the reproductive and seed-filling stages and evaluate its impact on mung bean seed-filling duration and seed quality. Furthermore, we sought to identify the genetic traits associated with heat tolerance and susceptibility among the contrasting mung bean genotypes. This information holds the potential to greatly enhance breeding programs aimed at developing heat-tolerant mung bean varieties for sustainable production in regions prone to high temperatures.

## Materials and methods

### Raising of plants

**Figure 1:**
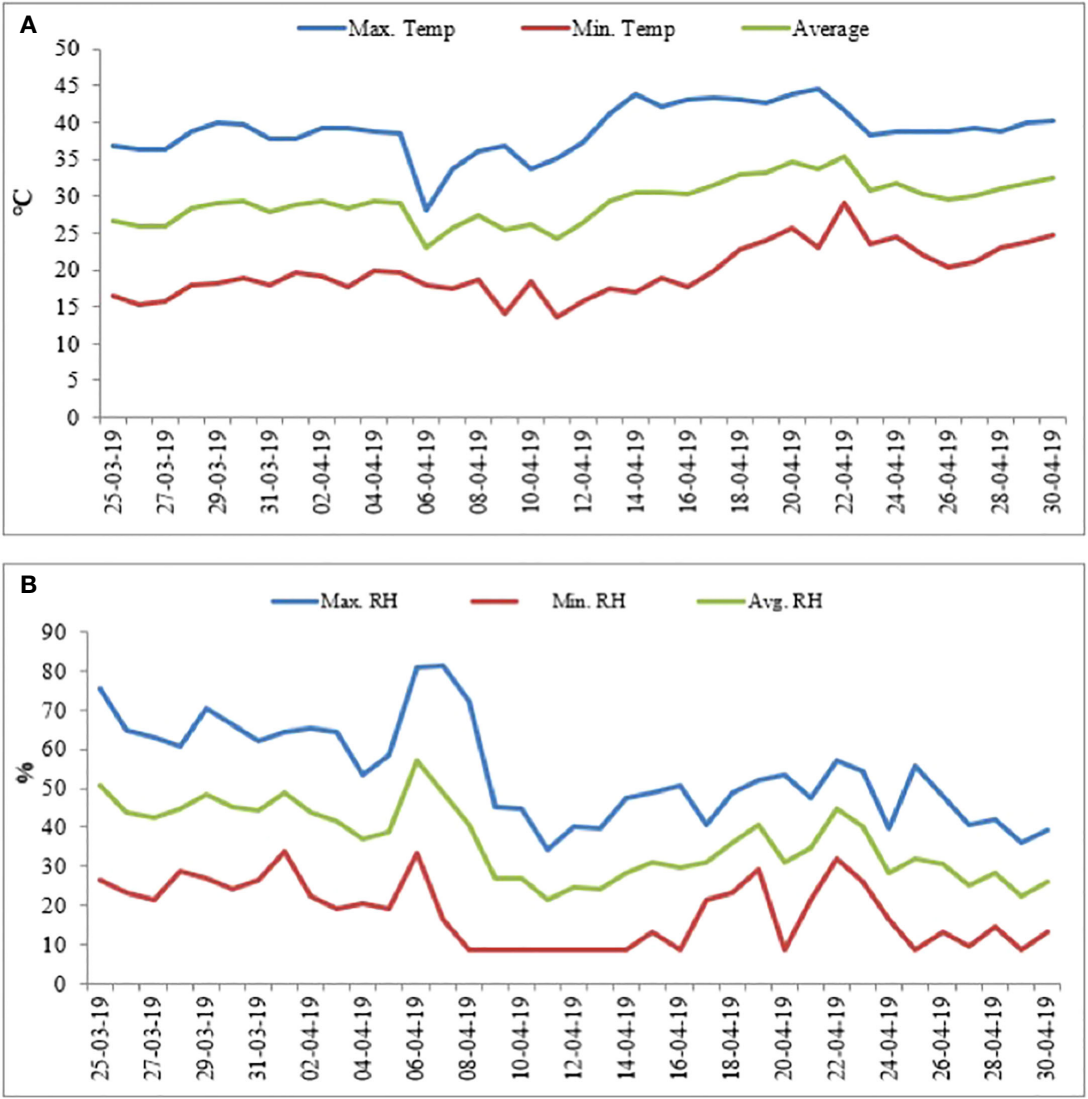
Weather data (2019) showing day/night temperatures **(A)** and relative humidity **(B)** from sowing until the start of seed filling.

Mung bean genotypes (heat-tolerant [HT: EC 693369] and heat-sensitive [HS: KPS1]) procured from the World Vegetable Center (ICRISAT, India) were raised in pots (8 kg capacity) containing air-dried soil (loam; pH 7.1, available N, P, and K of 54, 43, and 158 kg ha^−1^, respectively), sand, and farmyard manure (2:1:1 (v/v) ratio). Before sowing, the seeds were inoculated with a suitable Rhizobium strain (2.0 g kg^−1^ seeds). Four seeds were planted in each pot on 20/03/2019 and thinned to two seedlings per pot after emergence. The plants were grown in an outdoor natural environment with average day/night temperatures of 36°C/24.3°C, relative humidity (RH) of 50.3%/22.3% max/min, and light intensity of approximately 1,500 μmol m^−2^ s^−1^–1,700 μmol m^−2^ s^−1^ (**Figures 1A, B**) until the beginning of pod set (approximately 40 days after sowing). At this time, half of the pots (five pots in three replications; 5 × 3 = 15 pots per genotype) were transferred to a growth chamber set at day/night temperatures of 35°C/25°C, RH of approximately 65%, and light intensity of around 500 μmol m^−2^ s^−1^ (control treatment), and the other half (five pots in three replications; 5 × 3 = 15 pots per genotype) were transferred to a growth chamber set at day/night temperatures of 42°C/30°C, RH of approximately 65%, and light intensity of around 500 µmol m^−2^ s^−1^ (heat stress treatment), where they remained until maturity. The temperatures for the control (35°C/25°C) and heat stress (42°C/30°C) treatments were based on our previous studies. The experiment had a randomized block design with two genotypes (HT and HS), two treatments (control and heat stress), and three replications.

### Stress injury to leaves

Leaves were collected from the control and heat-stressed plants after 5, 10, 15, 20, 25, and 30 days of pod filling to assess membrane damage using an electrolyte leakage assay (Lutts et al., 1996). Fresh leaves from the topmost branches were washed with deionized water to remove contaminants and surface-attached electrolytes. The leaf tissue was placed in vials containing 10 mL of deionized water and incubated for 24 h at 25°C on a rotary shaker. The electrical conductivity was determined using a conductivity meter.

### Leaf water status

Relative leaf water content (RLWC) was measured on the topmost leaves by determining the fresh, dry, and turgid weights according to the method described by Barrs and Weatherley (1962). Stomatal conductance was measured on the same leaves using a leaf porometer, as described previously (Kaushal et al., 2013).

### Photosynthetic efficiency

Fresh leaves were extracted in 80% acetone and assayed spectrophotometrically at 645 and 663 nm to measure the leaf chlorophyll concentration using the method described by Arnon (1949). Chlorophyll fluorescence, in terms of PSII activity, was measured on the same leaves as chlorophyll fluorescence, using a modulated chlorophyll fluorometer (OS1-FL, Opti-Sciences, Tyngsboro, MA, USA) at 11:00 h, as previously described (Kaushal et al., 2013).

### Enzyme assays

Enzyme activity profiles (soluble starch synthase, sucrose synthase, and acid invertase) were determined using fresh seeds collected at various stages after pod filling (5, 10, 15, 20, 25, and 30 days) from pods on the uppermost branches of the control and heat-stressed plants. Seeds were collected randomly from pods in triplicates per genotype. Seeds (500 mg) were homogenized in ice-cold extraction medium containing HEPES/KOH buffer (200 mM, pH 7.8), 1% (w/v) polyvinylpyrrolidone (PVP), 3 mM EDTA Na2.2H2O, 10 mM dithiothreitol (DTT), and 3 mM magnesium acetate. The homogenate was centrifuged at 4°C for 20 min (10,000 × g), and the supernatant was used to assay enzymes and proteins. The enzyme extract was desalted and assayed for sucrose synthase, soluble starch synthase, and acid invertase according to the methods described by Xu et al. (1996) and Sung et al. (1989).

### Seed reserves

Various seed reserves (starch, sucrose proteins, fats, and storage proteins) were determined in mature seeds collected from control and heat-stressed plants at maturity. Starch and soluble sugars were extracted with 30% (v/v) perchloric acid and 95% (v/v) ethanol, respectively, and determined according to the method of Dubois et al. (1956), using glucose (Sigma D9434; Sigma, WI, USA) as a standard. Crude proteins, crude fats, and mineral nutrients were analyzed using standard AOAC procedures.

Storage proteins were sequentially isolated as fractions, according to the method described by Triboi et al. (2000). The seeds were homogenized into wholemeal flour, and the samples were stirred continuously for 60 min on a magnetic stirrer. The protein fractions (soluble and insoluble) were separated by centrifugation at 8,000*g* for 30 min at an appropriate extraction temperature. Albumins and globulins were extracted at 4°C with 25 mL of sodium phosphate buffer (0.05 M; pH 7.8) and NaCl (0.05 M), respectively. Prolamins were extracted from the previous pellet using 25 mL of 70% (v/v) ethanol at 20°C. Glutelins were extracted from the earlier pellet with 25 mL of 20 g L^−1^ sodium dodecyl sulfate (SDS), 2% (v/v) 2-mercaptoethanol (2-SH), and 0.05 M tetraborate buffer (pH 8.5) at 20°C. Glutelin was recovered from the supernatant by centrifugation. The protein concentration in each fraction was estimated according to the method described by Lowry (1951).

### Seed growth rate and seed-filling duration

The seed growth rate from five pods plant^−1^, tagged at the start of pod filling (pod size ~1 cm) and followed until physiological maturity, was determined. Seeds were measured for their dry weight at two stages; 7 days after the onset of pod filling and at physiological maturity. Measuring the difference between the dry weights at the two stages and dividing by the number of days to reach physiological maturity revealed a seed-filling rate day^−1^. Many pods, set on the same date, were marked to record these traits. Pods of the same size and dimension were selected to record these observations. The seeds harvested from the pods were oven-dried for 5 days at 45°C, and their dry weights were recorded. The number of days to complete seed filling for the tagged pods was recorded to calculate the seed-filling duration.

### Yield parameters

To measure yield traits, 10 plants from each genotype were examined for each treatment. The seed weight, number of seeds number plant^−1^, and individual seed weight were recorded for each case.

### Statistical analysis

Observations were replicated three times, with the data analyzed for means and standard errors. ANOVA was conducted, with the least significant values (LSD) calculated (P < 0.05) using AGRISTAT software. Principal component analysis (PCA) and correlation coefficient determination were performed using the R software.

## Results

**Tables 1**, **2** show the mean sum of squares and genotype × treatment interaction significance for various traits, respectively.

**Table 1:** ANOVA values for the mean sum of squares for leaf traits, seed traits, and enzymes across the two mung bean genotypes under heat stress.

Leaf traits and seed traits.																
Source of variation	d.f	EL		Chl	ChlF	RLWC		gS		SFD		SFR		SSW	SNP	SWP
**Treatment**	1	55.8*		62.08*	0.032**	390.4*		59,004.1**		108.3		15.68*		81.4	3123.6*	3.63**
**Replication**	2	3.86		0.52	0.005	10.05		1,086.70		0.64		0.01		0.02	0.27	0.14
**Error**	2	2.01		0.46	0	0.37		42.6		8.1		0.28		13.4	33.7	0.008
Seed constituents																
Source of variation		d.f	Carb		Proteins		Fat		Suc		SS		SSS		AI	
**Treatment**		1	40,491.6*		8,392.5**		16**		101.6		732.6*		1,338,592**		406,640**	
**Replication**		2	5.2		0.38		1.1		0.52		98.9		289		292.3	
**Error**		2	846.5		45.29		0.002		6.8		19.1		18.16		860.10	

EL, electrolyte leakage; Chl, SPAD chlorophyll; ChlF, chlorophyll fluorescence; RLWC, relative leaf water content; gS, stomatal conductance; SFD, seed filling duration; SFR, seed filling rate; SSW, single seed weight; SNP, seed number plant^−1^; SWP, seed weight plant^−1^.

Carb, carbohydrates; Suc, sucrose; SS, sucrose synthase; SSS, soluble starch synthase; AI, acid invertase.

*Significance at P < 0.05; ** at P < 0.01.

**Table 2:** Analysis of variance (ANOVA) showing statistical significance in various traits measured in two mung bean genotypes across the treatments.

Traits	Genotype	Treatment	Interaction
Electrolyte leakage	**	**	*
Chlorophyll	**	*	*
Chlorophyll fluorescence	**	*	**
Relative leaf water content	**	*	*
Stomatal conductance	ns	**	**
Seed filling duration	**	**	*
Seed filling rate	**	**	*
Single seed weight	*	**	ns
Seed number plant^-1^	**	**	**
Seed weight plant^-1^	**	**	ns
Seed carbohydrates	**	**	**
Seed proteins	**	**	*
Seed fat	**	**	*
Seed sucrose	*	**	*
Seed sucrose synthase	**	**	ns
Seed soluble starch synthase	**	**	**
Seed acid invertases	**	**	**

*Significance at P < 0.05, **significance at P < 0.01, ns = non-significant.

### Heat stress injury to leaves

#### Membrane damage

**Figure 2:**
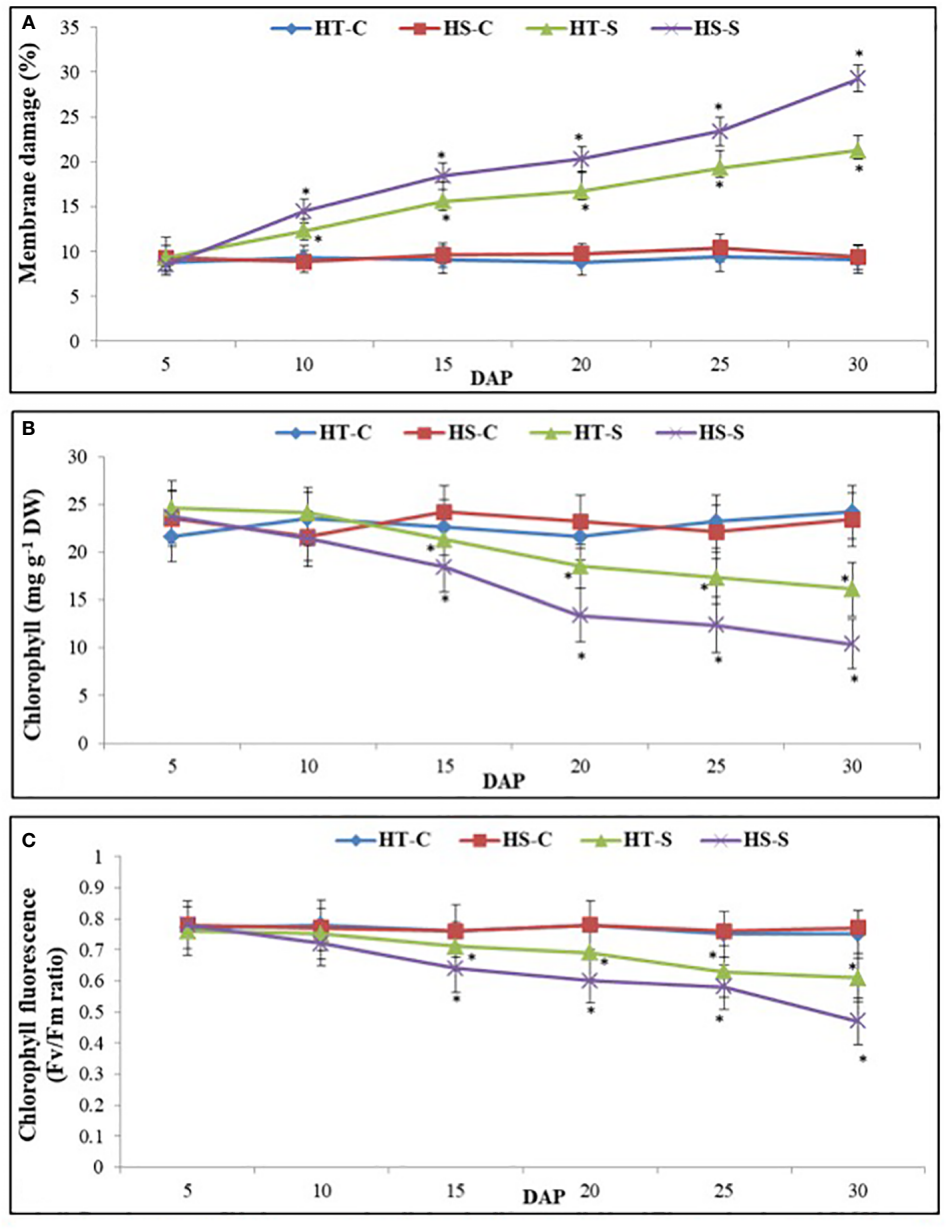
Membrane damage **(A)**, chlorophyll content **(B)**, and chlorophyll fluorescence **(C)** in control and heat-stressed (S) leaves of heat-tolerant (HT) and heat-sensitive (HS) genotypes on different days after pod filling (DAP). * indicate significance differences (P<0.05) between control and heat-stressed HT and HS genotypes at various DAP.

As measured by electrolyte leakage, the percentage of damage to leaf membranes (**Figure 2A**) from 5 to 30 days after pod filling (DAP)for the control plants was approximately 9% in the HT genotype and 11% in the HS genotype. However, the HS genotype under heat stress experienced 29% damage at 30 DAP compared with 21% damage in the HT genotype.

#### Chlorophyll concentration

The chlorophyll concentration (Chl) in control plants ranged from 21.6 mg g^−1^–24.2 mg g^−1^ DW in the HT genotype and 21.3 mg g^−1^–23.4 mg g–1 DW in the HS genotype (**Figure 2B**). However, the Chl concentration significantly decreased from 5 to 30 DAP under heat stress, with a 56% decrease in the HS genotype compared with a 34% decrease in the HT genotype.

#### Chlorophyll fluorescence

Chlorophyll fluorescence (ChlF) in the control plants ranged from 0.75 to 0.77 units (Fv/Fm ratio) in the HT genotype and 0.76–0.78 units in the HS genotype (**Figure 2C**). The ChlF levels significantly decreased from 5 to 30 DAP under heat stress, with a 39% decrease in the HS genotype and a 19% decrease in the HT genotype.

#### Leaf water status

**Figure 3:**
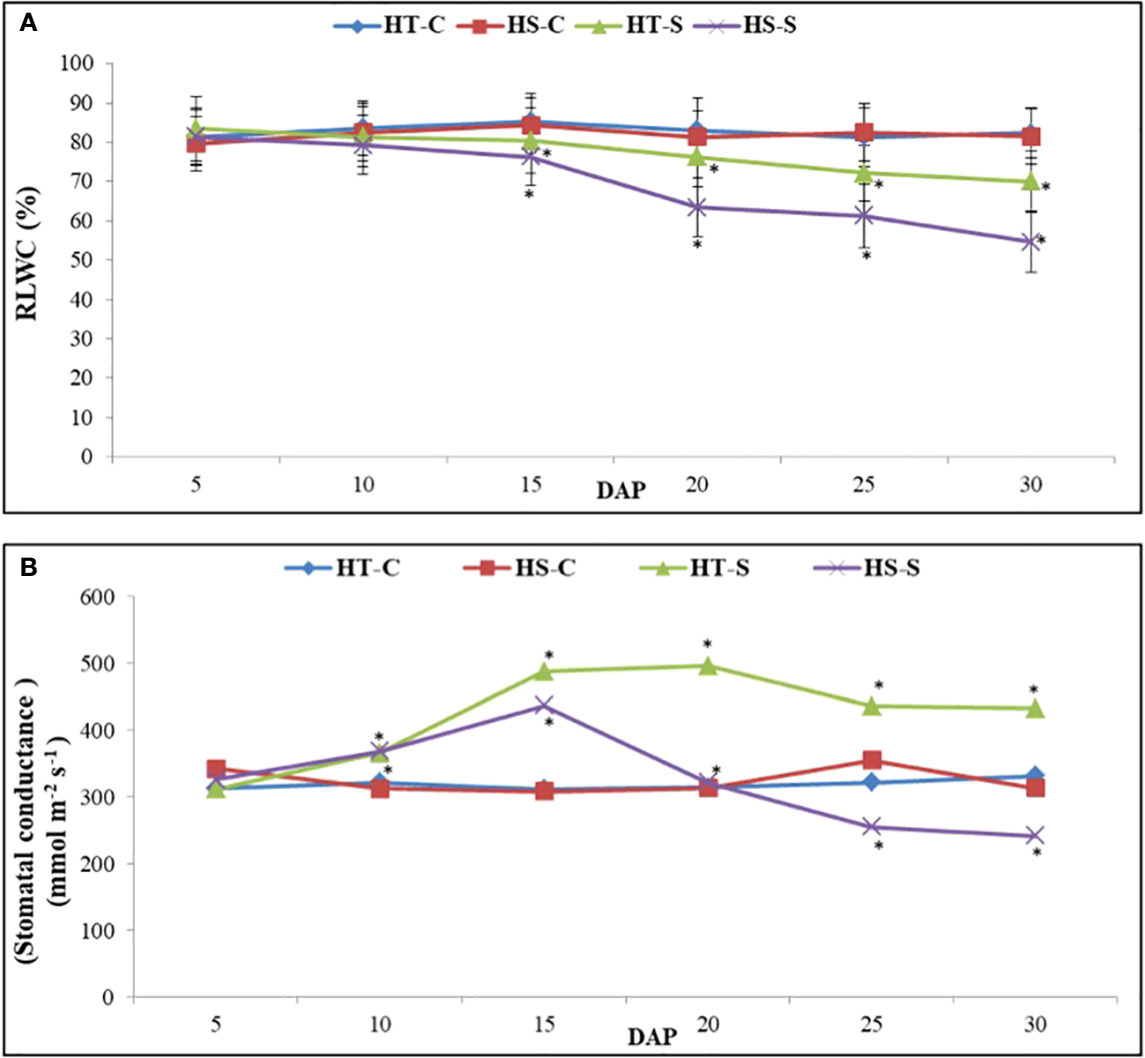
Relative leaf water content **(A)** and stomach condutance **(B)** in control and heat-stressed (S) leaves of heat-tolerant (HT) and heat-sensitive (HS) genotypes on different days after pod filling (DAP). * indicate significant differences (P<0.05) between control and heat-stressed HT and HS genotypes at various DAP.

Relative leaf water content (RLWC; **Figure 3A**) in the control plants ranged from 81.3% to 85.3% in the HT genotype and from 79.7% to 84.3% in the HS genotype. However, under heat stress conditions, the RLWC decreased significantly. Specifically, between 5 and 30 DAP, the RLWC in the HS genotype decreased to 54.5% whereas the HT genotype showed a higher RLWC value of 70.1%.

The stomatal conductance (gS) of the control plants in the HT genotype ranged from 311.3 mmol m^−2^ s^−1^ to 331.3 mmol m^−2^ s^−1^, and in the HS genotype, it ranged from 312 mmol m^−2^ s^−1^ to 325.6 l mmol m^-2^ s^−1^ (**Figure 3B**). Heat stress gradually increased gS in the HT genotype from 5 to 20 DAP, after which it decreased at 30 DAP. However, in the HS genotype, gS followed a similar trend but reached a much lower value at 30 DAP.

### Seed traits

The seed-filling rate of the control plants was 8.5 mg day^−1^ in the HT genotype and 6.8 mg day^−1^ in the HS genotype (**Table 3**). Under heat stress, the seed-filling rate significantly decreased by 32% and 20% in the HS and HT genotypes, respectively, compared with that of the controls.

**Table 3:** Various seed traits in control and heat-stressed mung bean genotypes.

Trait	Heat-tolerant		Heat-sensitive		LSD (P < 0.05)
	Control	Heat-stressed	Control	Heat-stressed	
Seed growth rate (mg day^–1^)	8.5 ± 0.54a	6.8 ± 0.48b	6.4 ± 0.61b	3.2 ± 0.53c	0.68
Seed-filling duration (days)	31.3 ± 1.7a	29.7 ± 1.5 b	25.6 ± 1.4c	16.5 ± 1.6d	1.6
Seed number (plant^–1^)	163.4 ± 5.1a	142.1 ± 4.7b	121.3 ± 5.9c	76.8 ± 4.2d	5.9
Seed weight (g plant^–1^)	5.78 ± 0.35a	4.66 ± 0.26b	4.02 ± 0.28c	2.41 ± 0.25d	0.31
Individual seed weight (g)	0.034 ± 0.004a	0.026 ± 0.003b	0.024 ± 0.004c	0.016 ± 0.003d	0.0046

Values represent mean ± SE (n = 3) along with LSD values (P < 0.05). Different letters in a row for a trait indicate significant differences (P < 0.05).

The seed-filling duration (SFD) of the control plants was 31.3 days in the HT genotype and 29.7 days in the HS genotype (**Table 3**). Under heat stress, relative to the control, SFD significantly decreased by 44% in the HS genotype to 16.5 days and 18% in the HT genotype to 25.6 days.

**Figure 4:**
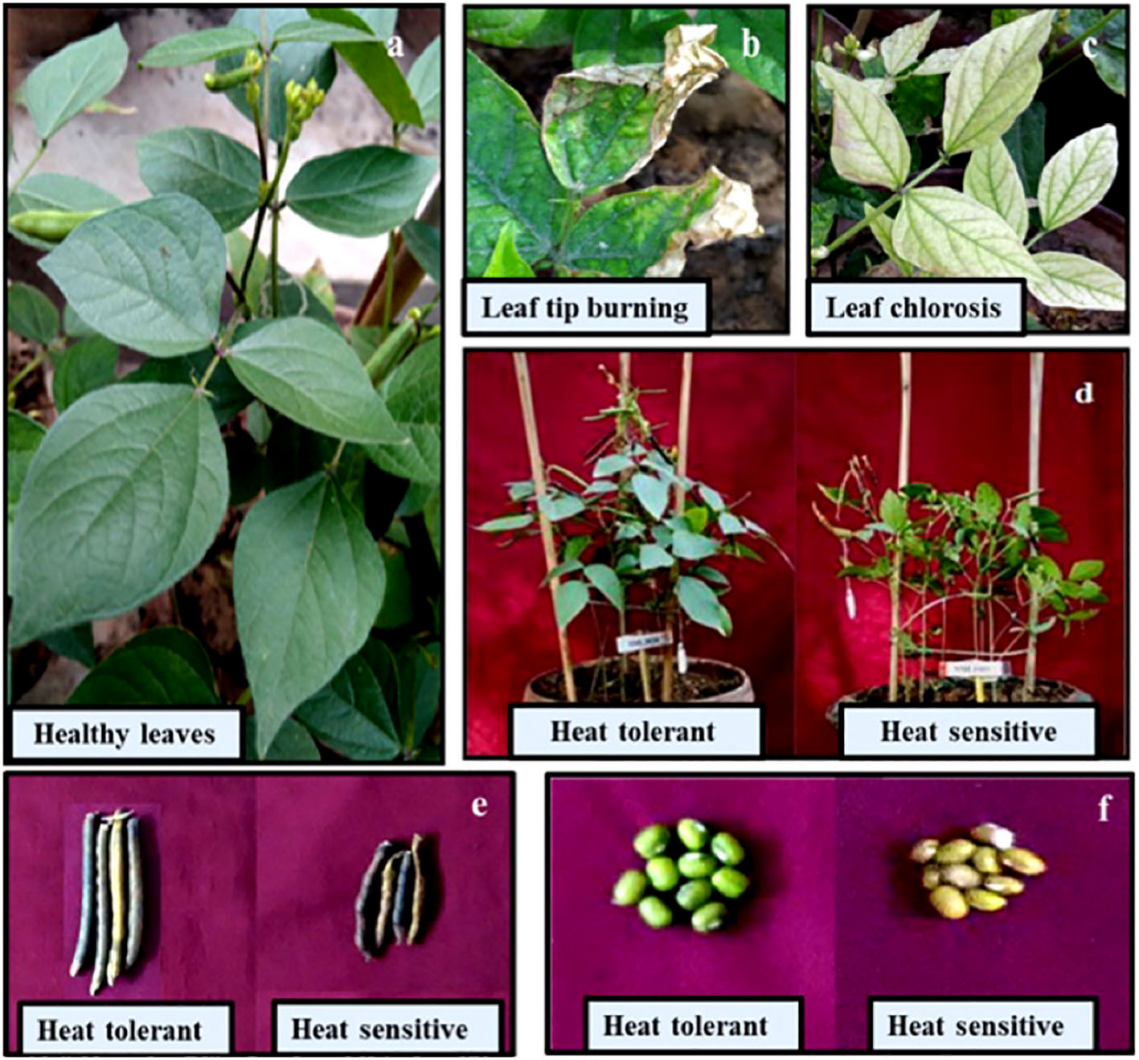
Adverse effects of heat stress: Leaves in control plants **(A)**, leaf burning **(B)**, chlorosis **(C)**, genotypes differences under heat stress **(D)**, pod size **(E)** and seed size **(F)**.

The control plants of the HT and HS genotypes had single-seed weights of 34.5 mg and 26.3 mg, respectively (**Table 3**). Heat stress significantly decreased the single-seed weight of the HS and HT genotypes by 32% and 23%, respectively, compared with their respective controls (see **Figure 4**).

Heat stress significantly reduced the seed number plant^−1^ (**Table 3**) by 36.6% in the HS genotype and by 13% in the HT genotype relative to the control plants (see **Figure 4**).

Seed weight plant^−1^ (**Table 3**) significantly decreased under heat stress by 40% in the HS genotype and 19.3% in the HT genotype compared with the control plants.

### Nutritional traits

Heat stress significantly decreased carbohydrate accumulation by 45% in the HS genotype and 24% in the HT genotype, relative to their respective controls (**Table 4**).

**Table 4:** Various seed constituents of control and heat-stressed mung bean genotypes.

Seed constituents (g kg^–1^)	Heat-tolerant		Heat-sensitive		LSD (P < 0.05)
	Control	Heat-stressed	Control	Heat-stressed	
Carbohydrates	603.4 ± 9.4a	560.4 ± 8.86b	483.6 ± 9.3c	310.4 ± 9.2d	12.3
Proteins	246.5 ± 8.7a	213.5 ± 9.4b	183.4 ± 8.4c	110.4 ± 8.4d	11.3
Fats	10.09 ± 1.7a	9.7 ± 1.2b	7.6 ± 1.3c	4.3 ± 1.2d	1.6
Albumins	89.6 ± 6.4a	81.3 ± 6.1b	64.5 ± 5.8c	44.6 ± 5.3d	6.8
Globulins	510.4 ± 11.3a	489.2 ± 10.3b	413.4 ± 9.5c	265.4 ± 8.6d	7.8
Glutelins	187.4 ± 8.5a	157.5 ± 9.4b	123.5 ± 8.4c	90.4 ± 8.5d	9.5
Prolamins	25.3 ± 3.5a	21.3 ± 2.3b	18.7 ± 2.1c	11.3 ± 2.5d	3.6

Values represent mean ± SE (n = 3) along with LSD values (P < 0.05). Different letters in a row for a trait indicate significant differences (P < 0.05).

Heat stress significantly decreased seed protein content by 48.4% in the HS genotype and 27% in the HT genotype, compared with the control plants (**Table 4**).

Heat stress significantly decreased seed fat content by 55% in the HS genotype and 30% in the HT genotype, relative to their respective controls.

### Seed storage proteins

Heat stress significantly decreased seed albumins, globulins, prolamins, and glutelins in the HS and HT genotypes (**Table 4**) by 45% and 28%, 47% and 19%, 42.6% and 34%, and 46.9%, and 26.3%, respectively, compared with the control plants.

### Minerals

The HS genotype had significantly greater reductions in mineral accumulation (**Table 5**) under heat stress compared with the control, particularly for iron and zinc, than the HT genotype. The HS genotype also showed greater reductions in calcium, magnesium, potassium, and phosphorus compared with the controls, than the HT genotype.

**Table 5:** Minerals in seeds of control and heat-stressed mung bean genotypes.

Minerals (g kg^–1^)	Heat-tolerant		Heat-sensitive		LSD (P < 0.05)
	Control	Heat-stressed	Control	Heat-stressed	
Calcium	0.114 ± 0.043a	0.0097 ± 0.046b	0.0867 ± 5.2b	0.0513 ± 0.038d	0.053
Iron	0.0083 ± 0.002a	0.0076 ± 0.002b	0.0062 ± 0.0013c	0.0046 ± 0.0012d	0.004
Phosphorus	1.43 ± 0.31a	1.34 ± 0.21b	1.18 ± 0.26c	0.956 ± 0.18d	0.21
Potassium	1.32 ± 0.26a	1.23 ± 0.19b	1.06 ± 0.18c	0.76 ± 0.19d	0.22
Magnesium	0.194 ± 0.04a	0.168 ± 0.05b	0.113 ± 0.05c	0.089 ± 0.06d	0.09
Zinc	0.0036 ± 0.0011a	0.0028 ± 0.0013b	0.0021 ± 0.0014c	0.0013 ± 0.0011d	0.0016

Values represent mean ± SE (n = 3) along with LSD values (P < 0.05). Different letters in a row for a trait indicate significant differences (P < 0.05).

### Sucrose metabolism in seeds

#### Sucrose

**Figure 5:**
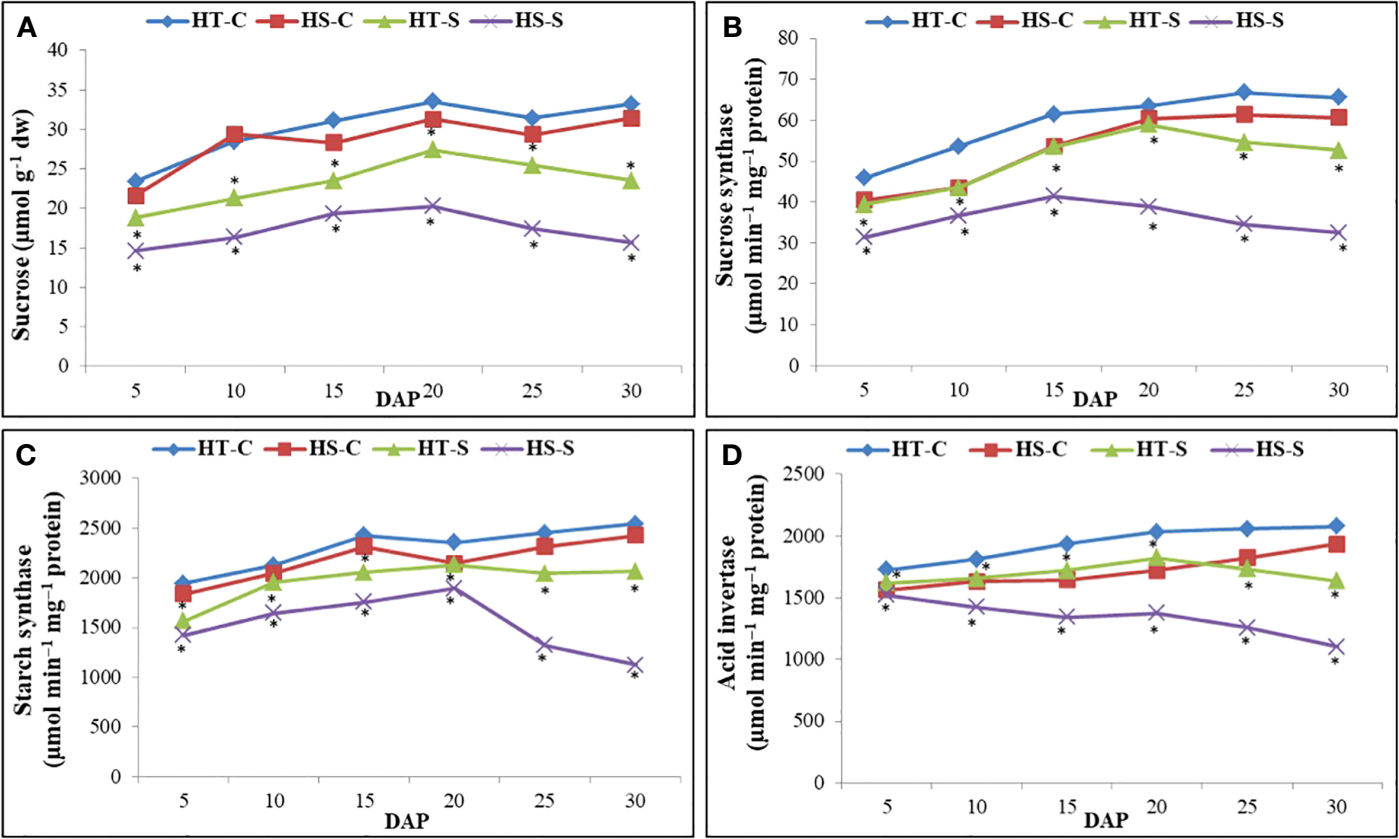
Sucrose content **(A)**, sucrose synthase activity **(B)**, starch synthase activity **(C)** and acid invertase activity **(D)** in control and heat-stressed (S) seeds of heat-tolerant (HT) and heat-sensitive (HS) genotypes on different days after pod filling (DAP). *indicate significant differences (P<0.05) between control and heat-stressed HT and HS genotypes at various DAP.

Heat stress decreased sucrose accumulation from 20 to 30 DAP, more so in the HS genotype than in the HT genotype at 30 DAP (**Figure 5A**).

#### Sucrose synthase

Heat stress increased sucrose synthase (SS) activity from 5 to 20 DAP in the HT genotype and from 5 to 15 DAP in the HS genotype (**Figure 5B**). However, SS activity significantly decreased in the HS genotype from 15 DAP onward. At 30 DAP, the heat-stressed HT genotype had significantly higher SS activity (52.6 µmol min^−1^ mg^−1^ protein) than the heat-stressed HS genotype (32.5 µmol min^−1^ mg^−1^ protein).

#### Soluble starch synthase

The heat-stressed HT and HS genotypes increased soluble starch synthase activity from 5 to 20 DAP but subsequently decreased. At 30 DAP, the heat-stressed HT genotype exhibited significantly higher soluble starch synthase activity than the heat-stressed HS genotype.

#### Acid invertases

Acid invertase activity increased in the heat-stressed HT genotype from 5 to 20 DAP but subsequently decreased (**Figure 5D**). In contrast, acid invertase activity decreased in the heat-stressed HS genotype from 5 to 15 DAP. At 30 DAP, the heat-stressed HT genotype exhibited significantly higher acid invertase activity than the heat-stressed HS genotype (**Figure 5C**).

### Correlations and PCA

**Figure 6:**
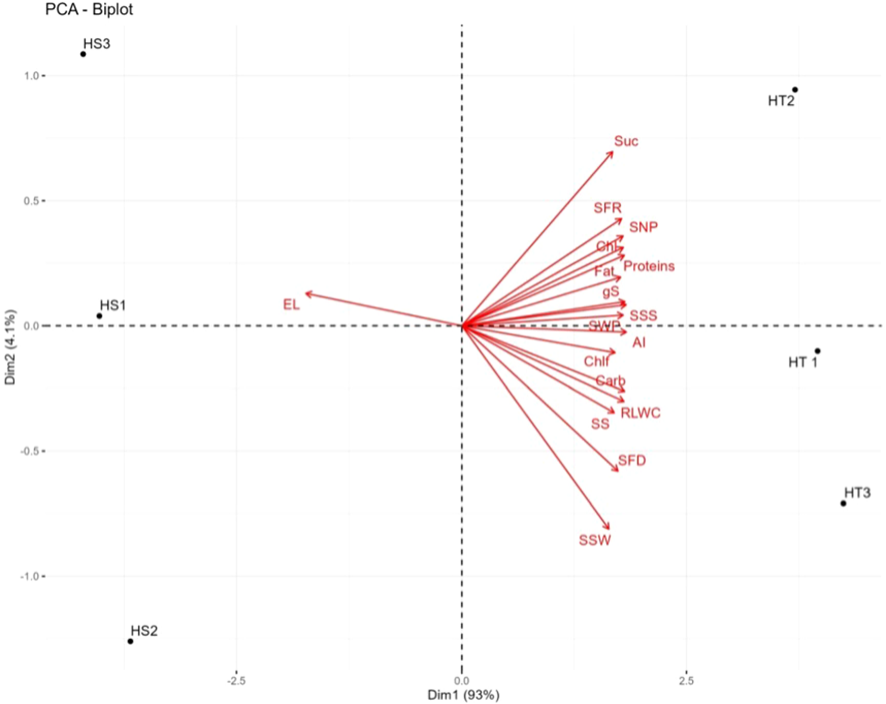
Principal component analysis (PCA; biplot) for heat tolerant and heat sensitive genotype under heat stress. 1. Suc-Sucrose, 2.SFR-Seed filling rate,3. SNP-Seed number per plant,4.Chl-chlorophyll content, 5.gS-stomatal conductance, 6.SSS-single seed weight,7. SWP-seed eaight per plant. 8. Chlf-chlorophyll fluorescence, 9. AI-acid invertase, 10. RLWC-relative leaf water content, 11. SS-sucrose synthase, 12. SFD-seed filling duration, 13. SSW-single seed weight, 14. EL-eloctrolyte leakage, 15.HT-Heat Tolerant, 16. HS-Heat Sensitive, HT1, HT2, HT3 are replicates of heat tolerant and HS1, HS2, HS3 are the replicates of heat sensitive genotypes.

Several leaf and quality-related traits were positively correlated with SFD, seed number plant^−1^, and seed weight plant^−1^ (**Table 6**). The PCA of the HT and HS genotypes revealed significant differences in 17 studied traits under heat stress, including leaf traits, seed traits, yield traits, and enzyme activities. The first two principal components (PC1 and PC2) explained 97.1% of the total variability, with PC1 contributing 93% and PC2 contributing 4.1% (**Figure 6**). Electrolyte leakage had the most negative contribution (–0.944) to PC1, whereas all other variables contributed positively. RLWC, stomatal conductance, seed weight per plant, carbohydrates, proteins, soluble starch synthase, and acid invertase were identified as the major contributors to PC1. Strong positive correlations were observed between soluble starch synthase activity and carbohydrate (0.93*) and sucrose (0.86*) content in seeds. Sucrose synthase activity was also positively correlated with sucrose (0.88*), soluble starch synthase (0.71*), and acid invertase (0.83*) activities in seeds. ()

**Table 6:** Correlation coefficients of various traits with yield traits under heat stress.

	Seed filling duration	Seed number plant^−1^	Seed weight plant^−1^
Electrolyte leakage	-0.92*	-0.90*	-0.97**
Chlorophyll	0.87*	0.95**	0.97**
Fv/Fm	0.91*	0.91*	0.84*
Stomatal conductance	0.92**	0.93**	0.95**
RLWC	0.95**	0.95**	0.95**
Seed filling duration (SFD)	1.00	0.87*	0.92**
Seed filling rate (SFR)	0.85*	0.94**	0.94**
Single seed weight	0.94**	0.78	0.88*
Seed number/plant	0.87*	1.00	0.94**
Seed weight/plant	0.91*	0.94**	1.00
Seed carbohydrates	0.92**	0.84*	0.91*
Seed protein	0.89*	0.81*	0.81*
Seed fat	0.88*	0.92**	0.84*
Seed sucrose	0.75	0.92**	0.89*
Seed sucrose synthase (SS)	0.91*	0.88*	0.85*
Seed soluble starch synthase (SSS)	0.93**	0.92*	0.92**
Seed acid invertase (AI)	0.81*	0.83*	0.82*

*Denotes significant at 5% and ** denotes significant at 1%.

## Discussion

Our investigation focused on the impact of heat stress on seed filling in two contrasting mung bean genotypes. Studies have shown that heat stress adversely affects pod set and reduces seed number in mung bean (Sharma et al., 2016; Van Haeften et al., 2023). Our results showed that heat stress decreased seed number plant^−1^ in the HT and HS genotypes, likely due to a reduction in filled pods, similar to observations in other crops such as wheat (Balla et al., 2019), maize (Niu et al., 2021), lentil (Choukri et al., 2020), pea (Mohapatra et al., 2020), urdbean (Chaudhary et al., 2022a), and chickpea (Devi et al., 2022). Heat stress also significantly reduced seed weight and size, possibly due to decreased seed-filling rate and duration (Sarkar et al., 2021). The accelerated maturation of legume plants under heat stress could also hinder seed filling, decreasing seed weight (Gaur et al., 2015).

Studies on various crops, including maize (Wilhelm et al., 1999), rice (Begcy et al., 2018), mung bean (Bhardwaj et al., 2023), field pea (Sharma et al., 2023), and chickpea (Devi et al., 2022), have documented the adverse effects of heat stress on seed size. Heat stress disrupts seed development by impeding the transport and utilization of molecules and minerals, including carbohydrates such as sucrose, which serve as precursors for protein and fat synthesis (Kaushal et al., 2013). The leaves of heat-stressed mung bean plants showed chlorosis and necrosis, resulting in poor photosynthetic efficiency in both the genotypes. These changes correlated with significant reductions in leaf water status (RLWC) and stomatal conductance, especially in HS genotypes. High temperatures and photooxidation can damage leaf tissues and membranes, leading to the loss of chlorophyll pigments (Ibrahim, 2011; Chen et al., 2012 **;** Prasad et al., 2017). Consequently, sucrose production declines, limiting its availability and transport to the developing seeds (Weber et al., 1997; Weschke et al., 2000; Asthir et al., 2012). Reduced photosynthetic ability can impede seed filling in legumes, as has beeb reported in chickpea (Gaur et al., 2015). Moreover, heat stress adversely affects sucrose transport more than photosynthesis, further inhibiting sucrose synthesis (Asthir et al., 2012; Phan et al., 2013; Liu et al., 2019).

Seeds consist primarily of carbohydrates, proteins (including storage proteins), lipids, and minerals. These organic compounds originate from precursor molecules transported from the leaves to seeds. Invertases are essential for releasing glucose and fructose from imported leaf sucrose, with glucose involved in starch synthesis during seed development. However, under heat stress, the activities of the enzymes responsible for sucrose synthesis and utilization (sucrose synthase and acid invertases, respectively) and starch synthesis (soluble starch synthase) notably diminished in this study, decreasing carbohydrate accumulation in seeds. Similar adverse effects on enzymes involved in carbohydrate and protein synthesis have been reported in various crops, including chickpea (Kaushal et al., 2013), lentil (El Haddad et al., 2021), common bean (Soltani et al., 2019), and wheat (Zhao et al., 2008). We found that the HS genotype had more pronounced inhibition of sucrose synthase, acid invertase, and soluble starch synthase than the HT genotype, resulting in a greater reduction in seed size, possibly due to the decreased import of sucrose into seeds. Reduced starch synthesis in mung bean seeds further hindered their development, possibly due to the low acid invertase activity decreasing the availability of glucose precursors. Similar observations have been reported in heat-stressed maize (Wilhelm et al., 1999; Yang et al., 2018), wheat (Liu et al., 2011), and lentil (Sita et al., 2018). Sucrose synthase and starch synthase activities increased from 5 to 15 DAP in both mung bean genotypes but subsequently decreased, more so in the HS genotype than the HT genotype, similar to the patterns observed in the grain of heat-stressed maize (Yang et al., 2018) and ‘basmati’ rice (Ahmed et al., 2015).

Heat stress significantly reduced grain protein content, including storage proteins, in mung bean, indicating a lack of sufficient precursors and inhibition of biosynthetic enzymes (Triboi et al., 2000). Similar reductions in grain protein content in high-temperature environments have been reported in other crops such as wheat (Wilhelm et al., 1999) and lentil (Sita et al., 2018). Interestingly, heat-stressed maize experienced an increase in protein content due to the activation of related enzymes (Yang et al., 2018). Understanding the mechanisms underlying the decreased protein synthesis in heat-stressed mung bean seeds is essential for future studies. The decline in protein content can reduce grain nutritional quality, highlighting the need to explore strategies to mitigate the adverse effects of heat stress on crops.

The reduced fat synthesis in heat-stressed mung bean seeds is likely due to impaired photosynthesis, reducing acetyl-CoA production, as reported in the leaves of heat-stressed common bean (*Phaseolus vulgaris* L.) (Taiz and Zeiger, 2006). Similar studies have found that heat stress significantly decreases fat accumulation in mung bean seeds, consistent with observations in canola (*Brassica napus* L.) seeds exposed to high temperatures (Aksouh et al., 2001). However, the mechanism underlying the decrease in fat accumulation in heat-stressed mung bean seeds requires further investigation.

Heat-stress-induced leaf damage may have decreased the enzyme profiles in seeds, further disrupting photosynthesis due to the source–sink relationship interrupting seed filling. Positive correlations were observed between soluble starch synthase activity and carbohydrate content in mung bean seeds, indicating the significance of this enzyme in controlling carbohydrate synthesis during storage. Similarly, positive associations occurred between sucrose synthase activity and sucrose, sucrose synthase and soluble starch synthase, and sucrose synthase and acid invertase activity, suggesting that sucrose synthase regulates sucrose metabolism and starch synthesis in mung bean seeds. Our findings corroborate those of Fan et al. (2019), who emphasized the importance of these enzymes in carbohydrate storage. These correlations highlight the potential of targeting these enzymes to enhance mung bean yield and quality under heat stress conditions.

Incorporating genotypes with diverse heat tolerance in this study captured a broad range of responses to high-temperature stress and enhanced our understanding of the underlying mechanisms involved. The PCA revealed significant differences between HT and HS genotypes across 17 traits, including leaf traits, seed traits, yield traits, and enzyme activities. The strong negative impact of electrolyte leakage on PC1 highlighted its pivotal role in determining heat sensitivity in mung bean genotypes. Conversely, positive contributions to PC1, including RLWC, stomatal conductance, seed weight per plant, carbohydrates, proteins, soluble starch synthase, and acid invertase, likely play integral roles in determining heat tolerance. These findings underscore the significance of maintaining appropriate plant hydration, managing stomatal conductance, and ensuring adequate levels of carbohydrates, proteins, and enzymes, such as soluble starch synthase and acid invertase, in developing HT mung bean genotypes. This knowledge can aid in identifying potential markers for selecting heat-tolerant genotypes and developing heat-resistant mung bean varieties. Previous studies have demonstrated that heat-tolerant genotypes often exhibit superior leaf traits, including membrane integrity (chickpea; Kumar et al., 2013; Devi et al., 2022), leaf water content (urdbean; Chaudhary et al., 2022a), chlorophyll levels (lentil; Sita et al., 2017), photosynthetic activity (mung bean; Sharma et al., 2016), and yield traits (Sita et al., 2017; Chaudhary et al., 2022b; Devi et al., 2022; Devi et al., 2023).

## Conclusions

This study revealed that heat stress significantly impacts the yield and quality of mung bean seeds during the seed-filling phase. Heat stress damages leaves and seeds, reducing seed numbers and weight and altering seed composition, encompassing carbohydrates, proteins, lipids, and minerals. Reductions in these crucial constituents can compromise seed nutritional value, posing potential risks to food security. Our investigations revealed that the two mung bean genotypes responded differently to high-temperature stress. The HT genotype (EC 693369) exhibited greater resilience to heat stress and maintained optimal leaf and seed functions at elevated temperatures, whereas the HS genotype (KPS1) experienced considerable damage to various cellular and enzymatic attributes. These findings suggest that the HT genotype could be the preferred choice for cultivation in regions susceptible to heat stress, as it can sustain higher yields and seed quality. These results underscore the detrimental effects of high-temperature stress on mung bean seed yield and quality, emphasizing the need to develop HT cultivars to ensure food security in areas prone to heat stress. Furthermore, this investigation emphasizes the importance of understanding the physiological mechanisms underlying heat stress responses in plants to develop effective strategies to alleviate the unfavorable effects of heat stress on crops.

## Data availability statement

The original contributions presented in the study are included in the article/supplementary material. Further inquiries can be directed to the corresponding authors.

## Author contributions

MP and AB: conducting the experiments, analysis, compilation of findings, writing, UJ: statistical analysis and interpretation, BH: provided seeds, editing inputs, PVVP, KDS, and KS: review and editing, HN and KS: conceptualization, final review, and editing. All authors contributed to the article and approved the submitted version.
